# Sociodemographic and Socioeconomic Factors Influencing the Body Mass Composition of School-Age Children

**DOI:** 10.3390/ijerph191811261

**Published:** 2022-09-07

**Authors:** Rafał Baran, Joanna Baran, Justyna Leszczak, Agnieszka Bejer, Justyna Wyszyńska

**Affiliations:** 1 Institute of Health Sciences, Medical College, University of Rzeszów, 35-959 Rzeszów, Poland; 2 SOLUTION-Statistical Analysis, 35-120 Rzeszów, Poland; 3 Natural and Medical Center for Innovative Research, 35-310 Rzeszów, Poland

**Keywords:** BMI, BFP, education status, place of residence, obesity

## Abstract

The purpose of the study was to evaluate the components of overweight, obesity, and body mass components among children aged 7 to 13 years against important sociodemographic factors. The analyses considered 315 school-age children from 7 to 13 years of age (164 boys and 151 girls). Each subject was assessed for body weight and height, body weight category, and main sociodemographic factors. Body mass components of body mass (body-fat percentage (BFP), muscle tissue, fat-free mass (FFM), and total body water (TBW) levels) were evaluated using the electrical bioimpedance method (BIA) and the TANITA 780 MC analyzer. A statistical analysis showed significant differences between the body composition of children living in cities in relation to children living in small towns and villages, and no significant differences were found between the results of children living in small towns and children living in villages. The presence of statistically significant differences between the values of the parameters of body composition of the studied children was demonstrated depending on the level of education of their fathers. The presence of statistically significant relationships between BMI of mothers and BFP of their children (*p* = 0.003), FFM (*p* = 0.003), muscle tissue (*p* = 0.001), and TBW (*p* = 0.001) has been demonstrated. The higher content of adipose tissue in children is strongly dependent on the higher BMI and body mass category of the mother, as well as the lower level of education of the father. The place of residence also significantly affects both the body fat content and the total body water content of body hydration. Living in the city is associated with better body composition.

## 1. Introduction

Overweight and obesity have become the civilization diseases of the 21st century [1,2,3,4,5]. Obesity is a complex multifactorial disease defined by excessive adiposity and is associated with an increased risk of many NCDs, including cardiovascular diseases (CVDs), 13 types of cancer, type 2 diabetes mellitus (DM2), and chronic respiratory diseases, including obstructive sleep apnea (OSA) [6,7,8]. Overweight and obesity affect almost 60% of adults and 1 in 3 children (29% of boys and 27% of girls) live with overweight or obesity [9,10]. Recent estimates suggest that overweight and obesity cause more than 1.2 million deaths in the WHO European Region every year, and is the fourth leading cause of death after high blood pressure, dietary risks, and tobacco—and corresponds to more than 13% of total deaths [11]. The World Health Organization (WHO) reports that in 2020, 39 million children under the age of 5 were overweight or obese. A high rate of obesity and overweight growth is evident among children and adolescents aged 5 to 19 years. In 2016, the occurrence of overweight and obesity increased dramatically in this group of children and young people by more than 18% (19% in boys, 18% in girls) [12]. In view of the constant increase in overweight and obesity, the WHO member states approved the project ‘No increase in childhood overweight by 2025’, which aims to provide a comprehensive plan for the implementation of maternal and child nutrition to prevent overweight and obesity. Therefore, prevention programs should be prioritized at the national and international level. Primary prevention is essential to reduce the incidence of obesity. Developing preventive programs is an investment in the future of children’s health [13,14].

Important factors influencing the occurrence of overweight and obesity are socioeconomic factors and the occurrence of depression. In the studies of Spinos et al., a significant indirect influence of socioeconomic status on BMI was found through mental stress and emotional eating. Lower socioeconomic status was associated with more stress and higher levels of suffering with more emotional eating, and higher emotional eating was associated with a higher BMI (b [SE] = −0.02 [0.01]; 95% CI: −0.04 to −0.01) [15]. The strong link between socioeconomic factors and obesity, coupled with new insights into the impact of socioeconomic disadvantages, implies that psychological and emotional stress is the primary link between socioeconomic disadvantages and weight gain. Children growing up in a dissonant family environment are particularly at risk, mainly due to the unfavourable socioeconomic situation of their parents, where they are exposed to parental frustration, discord in relationships, lack of support and consistency, negative belief systems, unmet emotional needs, and general insecurity [16]. Among the 143,603 children examined, the incidence of depression in obese children was 10.4%. Compared to children of normal weight, the chances of depression were 1.32-times higher (95% CI 1.17 to 1.50) in obese children. Among obese girls, the chance of depression was 1.44-times (95% CI 1.20 to 1.72) higher compared to girls of normal weight. There was no association between overweight children and depression (OR 1.04, 95% CI 0.95 to 1.14) or between the subgroups of obese or overweight men and depression (OR 1.14, 95% CI 0.93 to 1.41% and 1.08, 95% CI 0.85 to 1.37, respectively) [17].

This is why preventive programs based on a change in lifestyle in children and adolescents of school age are so important [18,19]. One of the very important components of lifestyle is undertaking physical activity. In the literature, authors increasingly state that school-age children spend too much time sitting [20,21,22]. According to WHO recommendations, children are encouraged to exercise at least 60 min a day through moderate to vigorous intensity aerobic exercise throughout the week, which will provide health benefits [23]. In multicentre ISCOLE studies that were conducted in children aged 9–11 from centers in 12 different countries (Australia, Brazil, Canada, China, Colombia, Finland, India, Kenya, Portugal, South Africa, the United Kingdom, and the United States), it was observed that the children had an average of 8.6 SED hours per day [24]. Sedentary lifestyle has a negative effect on the child’s body, so it is recommended to limit the recreational time spent in front of screens (e.g., TV, computer, electronic games) to no more than 2 h a day, with additional health benefits observed from shorter times spent in front of screens [25]. This is in line with recommendations from the Australian government [26] and the American Academy of Pediatrics [27].

Scientific reports on overweight and obesity in children from urban and rural areas are available in the literature. In studies among children aged 9 years from five voivodeships of Poland—Pomorskie, Opolskie, Wielkopolskie, Podkarpackie, and Mazowieckie—the prevalence of overweight and obesity in girls and boys in individual regions of the country (villages, cities with fewer than 100,000 inhabitants, and cities with more than 100,000 inhabitants) did not differ significantly [28]. However, studies in all voivodeships in Poland found significant differences in the prevalence of overweight and obesity between the regions analyzed. It was noticed that in the Małopolskie, Świętokrzyskie, Lubelskie, and Podkarpackie voivodships, the risk of overweight and obesity is lower than in the Mazowieckie voivodship. In the case of underweight, the Łódzkie and Podkarpackie voivodeships (<0.040) were areas of higher risk, and overweight (including obesity) was higher compared to underweight in most voivodships (15 of 16) [29]. From a meta-analysis of 49 studies with data from 323,420 children and adolescents aged 2 to 18 years from 26 countries. From 2000 to 2017, according to the IOTF criteria, the incidence of underweight showed an upward trend in eastern, northern, and southern Europe, where the occurrence of underweight ranged from 9.1% to 12.0%, from 4.1% to 6.8%, and from 5.8% to 6.7%, respectively. In western Europe, the incidence of underweight fell from 14.0% to 11.8%. There were no significant differences in terms of sex or age range [30].

An important aspect of the differentiation of the child’s body weight is the role of the family environment, including, in addition to the place of residence, the role of the education of the parents and their professional status [31,32,33,34]. They are the first teachers for children and role models. Therefore, we conducted an evaluation of the components of overweight, obesity, and body mass components (body-fat percentage (BFP), muscle tissue, fat-free mass (FFM), and total body water (TBW) levels) among children aged 7 to 13 years against important sociodemographic factors.

## 2. Materials and Methods

### 2.1. Study Design and Study Sample

Data for this study were collected during the period from November 2016 to December 2017. In the research project, 10 schools were randomly selected from all educational institutions in southeastern Poland, 4 schools from rural areas, 3 schools from small towns (less than 100,000 inhabitants), and 3 schools from a large city (over 100,000 inhabitants). More schools were randomly drawn in rural areas as they usually have fewer students. Forty pupils were randomly selected from eight of the schools and in two—38 and 30—due to the smaller number of pupils enrolled in the school.

The study involved 388 children from selected primary and secondary schools living in southeast Poland. After receiving written consent from parents, 322 children were qualified to participate. Four students were absent on the day of the study and three did not agree to participate in the study. Ultimately, the analyses considered 315 school-age children that were 7 to 13 years old (164 boys and 151 girls).

The criteria for inclusion in the study were the age of the child being between 7 and 13 years old, the consent of the parent and child to participate in the study, and place of residence being in a city, small town, or village.

The study’s exclusion criteria were epilepsy, implanted cardiac pacemaker, and girls being on their menstrual period on the day of the study.

### 2.2. Measurements

#### 2.2.1. Height

The height of the body was measured in cm. A Seca 225 height gauge was used to assess body height, which was performed three times to eliminate the possibility of a measurement error. The mean value of the three measurements was taken for analysis. During the examination, the child stood, without shoes, with their back to the measuring part of the height gauge. The device complies with Annex VI of Directive 93/42/EEC concerning medical devices [35,36].

#### 2.2.2. Body Composition

The TANITA 780 MC analyzer was used to evaluate the body weight and body mass composition of the subject. Body weight was measured in cm. The analyzer is certified according to the 93/42 EEC (EU standard for medical devices). Many researchers evaluated the reliability of the device in the pediatric population [37,38,39]. Independent research at several major universities (including Columbia University in New York City) has confirmed that in clinical settings, the Tanita Body Fat Monitor is accurate within ±5% of the institutional standard of body composition analysis-Dual Energy X-ray Absorptiometry (DEXA). The results of the Tanita Body Fat Monitor Series are repeatable to within ±1% variation when used under consistent conditions, and this device operates on the principle of bioelectrical impedance analysis (BIA). This method evaluates the electrical resistance, which is different for particular tissues of the body through which a low-intensity electrical current flow. The test procedure was as follows. First, the basic data of the patient, such as date of birth, sex, and body height, were entered into the analyzer. Then, after the beep, the patient was seated on the balance of the analyzer. After a while, when the device determined the body weight of the subject, the patient handed the tips with electrodes that enabled the evaluation of individual segments. The upper limbs were straight, slightly tilted toward the body. After another beep, the test was terminated, and the data were transferred to the computer program. On this basis, the body mass composition is differentiated and the body fat percentage (BFP), fat-free mass (FFM), and muscle, bone, and total body water (TBW) were determined. The percentage of total body water is the total amount of fluid in a person’s body expressed as a percentage of their total weight. The percentage of TBW percentage will tend to decrease as the percentage of body fat increases. An individual with a high percentage of body fat may fall below the average percentage of body water. As body fat is lost, the percentage of TBW should gradually move towards the typical range given above [40]. The composition of body mass, and especially the water content in the body, is of great interest among researchers, and the BIA method is a popular measurement method [41,42,43,44]. The percentages of BFP, FFM, muscle, and TBW were included in the analyses.

#### 2.2.3. Sociodemographic and Socioeconomic Data

The proprietary survey questionnaire evaluating sociodemographic factors consisted of two parts. The first part contained informed consent along with an explanation of all procedures that will be performed in each child examined and the factors indicated that exclude participation in the study. Furthermore, this part contained contact details for the people conducting the study if any of the participants had additional questions. The second part contained questions about sociodemographic factors (such as sex, age, place of residence of the subjects) as well as questions about the characteristics of the parents of the children concerned (e.g., age, body weight, height, education, professional status, BMI). A parent completed the questionnaire together with the child. The information about the parent came from the survey data, while the anthropometric data for the child were obtained during the examination.

### 2.3. Statistical Analysis

A statistical analysis of the material collected was performed in Statistica 10.0 by Statsoft, while a database and graphical results were compiled in Microsoft Excel. Both parametric tests and non-parametric tests were used to analyze the variables. The choice of a parametric test was conditioned by the fulfilment of its basic assumptions, i.e., the compliance of the distributions of the studied variables with the normal distribution, which was verified with the Shapiro-Wilk test. For all variables, descriptive statistics were calculated: mean, median, and standard deviation. The Student’s *t* test for independent variables or, alternatively, the non-parametric Mann-Whitney U test was used to assess the differences in the average level of a numerical feature in the two populations. A single-factor ANOVA (analysis of variance), or, alternatively, a non-parametric ANOVA Kruskal-Wallis test was used to assess the differences in the average level of a numerical feature in more than two populations. The correlation of two variables that did not satisfy the normality criterion was calculated by using a Spearman rank correlation coefficient. The level of statistical significance was *p* < 0.05.

### 2.4. Ethics

The study was conducted in accordance with the Declaration of Helsinki and was approved by the Ethics Committee or Ethics Committee of the University of Rzeszów (protocol code No. 18/12/2015 of 2 December 2015).

## 3. Results

The average age of the children studied was 10 ± 2 years. The height of the children studied was between 112 cm and 172 cm, with a mean of 141.81 ± 12.54 cm. The average body weight of the subjects was 35.82 ± 10.93 kg and ranged from 16.7 kg to 71.7 kg (Table 1).

Based on measurements of weight and body height, the BMI of the children studied was determined. The mean BMI of the subjects was 17.45 ± 3.17 kg/m^2^ and ranged from 11.5 kg/m^2^ to 29.9 kg/m^2^. In the percentile grids, the children examined were between the 0.1 and 99th percentiles. The average position of the children on the centile grid was estimated at about the 45th percentile (Table 1).

Based on the BMI values, 86 fathers (27.3%) had normal body weight, 176 fathers (55.9%) were overweight, and 53 fathers (16.8%) were obese. Of the mothers, 198 (62.9%) had normal body weight, 98 (31.1%) were overweight, 18 (5.7%) were obese, and one of the mothers (0.3%) was underweight (Table 2).

Most of the fathers had higher education (147—46.7%), followed by secondary education (141—44.8%) and vocational education (27—8.6%). Most mothers had higher education (170—54.0%), and the rest had secondary education (133—42.2%) and vocational education (12—3.8%). A total of 284 of the fathers (90.2%) and 246 of the mothers (78.1%) were professionally active (Table 2).

The analysis of the results started with an assessment of the effects of sociodemographic factors on the body mass composition of the children studied. No statistically significant relationship was demonstrated between the age of the studied children and the body mass composition (*p* > 0.05).

The presence of statistically significant differences between the values of the parameters of body composition of the girls and boys examined was demonstrated. BFP (*p* = 0.001), FFM (*p* = 0.001), muscle tissue (*p* = 0.018), and TBW were statistically different in two groups (*p* = 0.009). Girls had a higher BFP, while boys had a higher FFM, muscle tissue, and TBW (Table 3).

The presence of a statistically significant correlation between the values of the body composition parameters and the place of residence of the examined children was confirmed (*p* < 0.001) (Table 4). A post-hoc test showed significant differences between the body composition of children living in cities in relation to children living in small towns and villages, and no significant differences were found between the results of children living in small towns and children living in villages (Table 5).

The presence of statistically significant differences was demonstrated between the values of the body composition parameters of the studied children depending on the education level of their fathers. Statistically significant differences have been observed in three groups of fathers for parameters assessing BFP (*p* = 0.032), FFM (*p* = 0.032), muscle tissue (*p* = 0.003), and TBW (*p* = 0.004). However, differences in the body mass composition of the children were not demonstrated in relation to the education of the mother (Table 6).

A post-hoc test showed the presence of statistically significant differences in terms of BFP and FFM among children of fathers with higher and vocational education, while the results obtained among children of fathers with higher and secondary education and secondary and vocational education did not differ. In the case of muscle tissue content and TBW, statistically significant differences were observed between children of fathers with secondary and higher education compared to children of fathers with primary education, while the results obtained among children of fathers with higher and secondary education did not differ. Children of fathers with vocational education had the highest BFP, as well as the lowest FFM, muscle tissue, and TBW. Along with a higher level of education of the fathers of the studied children, FFM, muscle tissue, and TBW in the children’s bodies increased, while BFP decreased (Table 7).

There was no statistically significant relationship between the values of the parameters of body composition of the children studied and the professional activity of their fathers and mothers (active/inactive) (*p* > 0.05).

No statistically significant correlation was found between the values of the parameters of body composition of the studied children and their fathers’ BMI (*p*> 0.05).

The presence of statistically significant relationships between BMI of mothers and BFP of their children (*p* = 0.003), FFM (*p* = 0.003), muscle tissue (*p* = 0.001), and TBW (*p* = 0.001) has been demonstrated. The correlation between mothers’ BMI and BFP of the children studied was positive. This means that the higher the mothers’ BMI, the higher the BFP of their children. The correlations between mothers’ BMI and FFM, muscle tissue, and TBW in their children’s bodies were negative. This means that the higher the mothers’ BMI, the lower the FFM, muscle tissue, and TBW in their children’s bodies (Table 8).

There was no statistically significant relationship between the values of the parameters of the body composition of the children and the category of body weight of their fathers (*p* > 0.05).

However, the relationship between the values of the parameters of body composition of the children under study and the weight category of their mothers was statistically significant for all the parameters examined: BFP (*p* = 0.011), FFM (*p* = 0.011), muscle (*p* = 0.002), and TBW (*p* = 0.013) (Table 9).

A post-hoc test showed statistically significant differences in BFP, FFM, muscle, and TBW among children with mothers of normal weight and overweight. On the other hand, no statistically significant differences were found between children of normal weight and obese mothers and overweight and obese mothers. Children with overweight mothers compared to other children have been shown to have the highest BFP and the lowest FFM, muscle, and TBW in the body (Table 10).

## 4. Discussion

The associations between socioeconomic factors and excess weight measured by BMI have been extensively studied, but there is less evidence on the relationship between socioeconomic factors and body composition. Furthermore, the underlying mechanism and the role of socioeconomic factors as potential mediators of this relationship remain unclear. This study provided new evidence on the body composition of children aged 7–13 years from urban and rural Rzeszów, Poland, in relation to socio-environmental conditions. Overall, our findings suggest that among the determinants of body composition analyzed in children, significant ones include child sex, place of residence, father’s education, as well as mother’s BMI and the category of mother’s body weight. These socioeconomic indicators were strongly associated with childhood BFP, FFM, muscle content, and TBW. BFP was higher in girls compared to boys, and other body compositions (FFM, muscle%, and TBW) were higher in boys than in girls. Children in cities had a lower BFP content and higher muscle and TBW than children living in small towns or villages. Children of fathers with vocational education had the highest BFP, as well as the lowest FFM, muscle tissue, and TBW. Along with a higher level of education of the fathers, FFM, muscle tissue, and TBW in the children’s bodies increased, while BFP decreased. Mothers with a higher BMI had children with a higher content of BFP, lower FFM, muscle tissue, and TBW. Moreover, children with overweight mothers compared to children with normal-weight mothers have been shown to have the higher BFP and the lower FFM, muscle, and TBW. This finding is important due to the use of an objective assessment of the body composition of the children studied. Most of the evidence used BMI as a proxy for body adiposity because it is easier to determine, despite its limitations—such as not distinguishing BFP from FFM—and therefore it may under- or overestimate adiposity. For example, subjects with excessive muscle growth may show a high BMI without excess adiposity and may be mistakenly considered obese [45]. Our study used the foot-to-foot BIA method. According to Tyrrel et al., who compared BIA with DEXA in the population of children, there is a correlation between DEXA and BIA in the estimation of FFM, fat mass, and BFP. The authors concluded that BIA is a reliable and accurate method for determining BFP and other components of the child’s body [46]. Another study by Verney et al. indicated that the BIA method is an acceptable and reproducible alternative to DEXA to assess body composition in the pediatric population [38].

Body composition disorders are a growing problem nowadays. The fact that they are increasingly found in the younger population is particularly disturbing. According to evidence, there are many causes of body composition disorders, ranging from genetic and environmental factors to sociodemographic factors [47]. Increased BFP in childhood often leads to excess weight in adulthood and is related to a higher risk of developing many diseases, including hypertension, diabetes, dyslipidaemia, and cardiovascular disease, which in turn are related with an increased risk of premature mortality and disability. Orthopedic, neurological, pulmonary, gastroenterological, and endocrine conditions are more common in children with morbid obesity. Excess adiposity can affect a child’s immediate physical and mental health, educational achievements, quality of life, self-esteem, body image, and economic mobility [48,49].

Many studies have indicated an association between the body mass category of a parent and that of their child, but the underlying mechanisms are not well established [50,51,52]. Evidence suggested that BMI and obesity are strongly correlated among biological parent-child pairs, but there are no significant intergenerational associations in these health traits among adoptive parent-child pairs [50]. Our study showed that there was a significant association between maternal BMI and child body composition (*p* = 0.003 for BFP, *p* = 0.001 for muscle tissue). The correlation between mothers’ BMI and their children’s BFP was positive, that is, the higher the mothers’ BMI, the higher their children’s BFP was. The correlation between maternal BMI and child muscle tissue content was negative. The relationship between the body weight composition of the children and the weight category of their mothers was also significant. However, no significant correlation was found between the BMI of the fathers and the body weight categories in the child’s body weight composition. A meta-analysis of 23 studies indicated a significant association between parents and children who were overweight or obese (pooled OR = 1.97). The relationship between excess weight of parents and children was higher in Asia than in Europe and the Middle East, as well as higher in high-income countries than in middle- or low-income countries. Furthermore, a stronger correlation was observed between excess weight from parents and children when both parents were obese than when only one of the parents was obese [52].

The present study found a correlation between the father’s education and the child’s body composition. The children of fathers with vocational education had the highest BFP and the lowest muscle tissue. Along with a higher level of education of the fathers, the content of muscle tissue in the children’s bodies increased and the BFP decreased. There was no significant correlation between mother’s education and their children’s body composition. Gätjens et al., in a study of 4772 girls and boys aged 5–16 years, found that in all age groups, socioeconomic status (defined by ‘the level of parental education, classified as low, middle, or high’) was inversely associated with the BFP-SD score. The associations between socioeconomic status and the BFP-SD score were mediated by age-specific mediators, including parental BMI, parental smoking habits, media consumption, physical activity, and shared meals [53]. Another study by Fernández-Alvira et al. found that the relationship between parental education and child body composition was partially mediated by breakfast consumption, participation in sports, TV viewing, and computer use. Furthermore, a suppression effect was found for the consumption of sweetened beverages. No mediation effect was observed for active transportation and duration of sleep [54]. Mölenberg et al. indicated that children of less educated mothers that were aged 4 to 14 years, consumed more fast food and had an unhealthy food environment at home, which was associated with small increases in BMI [55].

The results of this study showed a significant association between the values of body composition parameters and the place of residence of the children studied. Compared to participants from small towns and villages, participants from a city had a higher content of muscle tissue and a lower BFP. The post-hoc test showed significant differences between the body composition of children living in a city in relation to children living in small towns and villages, and no significant differences were found between children living in small towns compared to children living in villages. A recent systematic review by Bridger Staatz et al. of 50 studies on socioeconomic position and body composition in childhood in high and middle income countries showed greater BFP and lower FFM in high-income countries, but lower BFP and lower FFM in middle-income countries. When evaluations of FFM indexed to height were used, there were no significant associations with socioeconomic position. In high-income countries, more evidence indicated relationships between disadvantaged socioeconomic position and higher BFP among girls compared to boys [56].

The present study has several strengths, mainly a relatively large sample size that includes children from both urban and rural areas. Another strength of the study is that body composition was measured using the BIA method, which has shown agreement with DEXA in the estimation of FFM, fat mass, and BFP [46]. However, Thivel et al. indicated that the precision of assessing body composition using BIA decreases with increasing obesity [37], which may be considered a limitation of our study. Another limitation is the cross-sectional nature; thus, the causal pathways underlying the observed relationships could hardly be detected. In addition, physical activity and diet were not adjusted in the analyses, which can compromise the results.

Our findings could be used to develop future public health policies to identify vulnerable subgroups and prioritize initiatives that aim to prevent and treat body composition abnormalities. Future studies that evaluate, explore, and compare these and other determinants of body composition, such as physical activity, sedentary time, or diet, will be of significant importance.

## 5. Conclusions

The sex of the respondents is a factor that strongly differentiates the composition of the respondents’ body weight. Girls are characterized by a higher content of adipose tissue, and boys are characterized by a higher content of lean tissue, including muscle tissue and water.

The place of residence of the respondents is another strong differentiating factor in relation to the components of body mass. Living in a city is associated with better mutual proportions of the individual components of the body. This may be related to a better level of knowledge about the correct body mass composition, however, this relationship should be thoroughly checked in future studies.

The level of education of the father influences the different content of the individual components of the body. The difference is especially visible between higher education (where the parameters of body mass composition were better) and vocational (where the parameters of body mass composition were worse).

Furthermore, a positive correlation was observed between the mother’s BMI and the child’s adipose tissue content. Children of healthy-weight mothers had a better body weight composition than children of overweight mothers.

The study conducted confirmed the significant role of the family environment and sociodemographic factors in the composition of body weight of children.

## Figures and Tables

**Table 1:** Characteristics of the study group—descriptive statistics.

	Descriptive Statistics							
	*n*	$\bar{x}$	Me	Min.	Max.	Q1	Q3	SD
**Children**								
Age [years]	315	10.00	10.00	7.00	13.00	8.00	12.00	2.00
Body height [cm]	315	141.81	142.00	112.00	172.00	132.00	151.00	12.54
Body mass [kg]	315	35.82	33.60	16.70	71.70	27.00	43.10	10.93
BMI [kg/m^2^]	315	17.45	16.90	11.50	29.90	15.20	19.30	3.17
BMI percentile [centile]	315	44.89	42.00	0.10	99.00	16.00	75.00	30.95
**Fathers**								
Age [years]	315	39.18	39.00	26.00	59.00	36.00	42.00	5.41
Body height [cm]	315	177.50	177.00	162.00	199.00	174.00	180.00	5.59
Body mass [kg]	315	86.06	85.00	60.00	120.00	80.00	93.00	11.57
BMI [kg/m^2^]	315	27.31	26.85	19.32	41.52	24.86	29.32	3.49
**Mothers**								
Age [years]	315	37.09	37.00	24.00	53.00	35.00	40.00	4.68
Body height [cm]	315	164.88	165.00	152.00	184.00	160.00	169.00	5.81
Body mass [kg]	315	64.89	65.00	43.00	90.00	58.00	70.00	9.74
BMI [kg/m^2^]	315	23.90	23.05	17.43	34.63	21.23	26.56	3.61

Max.—maximum value; Me—median; Min.—minimum value; *n*—number of subjects; Q1—first quartile; Q3—third quartile; SD—standard deviation.

**Table 2:** Body mass category on the basis of BMI as well as education and professional activity of parents.

	Children		Fathers		Mothers	
	*n*	%	*n*	%	*n*	%
**Body mass category based on BMI**						
Norm	246	78.1%	86	27.3%	198	62.9%
Overweight	28	8.9%	176	55.9%	98	31.1%
Obesity	11	3.5%	53	16.8%	18	5.7%
Underweight	30	9.5%	0	0.0%	1	0.3%
Total	315	100.0%	315	100.0%	315	100.0%
**Parent education**						
Professional			27	8.6%	12	3.8%
Medium			141	44.8%	133	42.2%
High			147	46.7%	170	54.0%
Total			315	100.0%	315	100.0%
**Professional activity of the parent**						
Professionally active			284	90.2%	246	78.1%
Professionally inactive			31	9.8%	69	21.9%
Total			315	100.0%	315	100.0%

*n*—numbers of subject; %—percent.

**Table 3:** Differences in body weight composition depending on the sex of the respondents.

The Parameters of the Child’s Body Composition	Girls			Boys			Z	*p*
	$\bar{x}$	Me	SD	$\bar{x}$	Me	SD		
**BFP (%)**	20.62	20.00	7.06	18.46	18.15	6.56	3.23	0.001
**FFM (%)**	79.38	80.00	7.06	81.54	81.85	6.56	−3.23	0.001
**Muscle (%)**	74.05	74.52	7.54	75.69	76.67	7.54	−2.36	0.018
**TBW (%)**	57.88	57.90	5.23	58.91	59.40	6.58	−2.58	0.009

BFP—body fat percentage; FFM—fat free mass; Me—median; *p*—probability value; SD—standard deviation; TBW—total body water; $\bar{x}$—average value; Z—Mann-Whitney U test value.

**Table 4:** Differences in the composition of body weight depending on the place of residence of the respondents.

The Parameters of the Child’s Body Composition	Place of Residence									H	*p*
	City			Small Town			Village				
	$\bar{x}$	Me	SD	$\bar{x}$	Me	SD	$\bar{x}$	Me	SD		
**BFP (%)**	15.10	14.40	6.98	22.37	21.00	5.46	21.02	19.90	5.83	63.03	<0.001
**FFM (%)**	84.90	85.60	6.98	77.63	79.00	5.46	78.98	80.10	5.83	63.03	<0.001
**Muscle (%)**	80.36	81.19	6.59	73.53	74.81	5.16	70.82	70.17	7.36	84.56	<0.001
**TBW (%)**	61.96	62.70	5.16	56.81	57.80	4.00	56.96	57.20	4.13	65.56	<0.001

BFP—body fat percentage; FFM—fat free mass; H—ANOVA Kruskal-Wallis test value; Me—median; *p*—probability value; SD—standard deviation; TBW—total body water; $\bar{x}$—average value.

**Table 5:** The post-hoc test in reference to the difference between the body build parameters in relation to place of residence.

Post-Hoc	BFP (%)			FFM (%)			Muscle (%)			TBW (%)		
	City	Small Town	Village	City	Small Town	Village	City	Small Town	Village	City	Small Town	Village
**Place of residence**												
City		<0.001	<0.001		<0.001	<0.001		<0.001	<0.001		<0.001	<0.001
Small Town	<0.001		0.313	<0.001		0.313	<0.001		0.110	<0.001		1.000
Village	<0.001	0.313		<0.001	0.313		<0.001	0.110		<0.001	1.000	

The values of the probability coefficient for the post-hoc test (multiple comparison test), BFP—body fat percentage, FFM—fat free mass, TBW—total body water.

**Table 6:** Differences in the composition of body weight depending on the parent’s education of the respondents.

The Parameters of the Child’s Body Composition	Parent Education									F	*p*
	Professional			Medium			High				
	$\bar{x}$	Me	SD	$\bar{x}$	Me	SD	$\bar{x}$	Me	SD		
**Fathers**											
**BFP (%)**	22.69	21.90	6.49	19.48	19.10	6.56	18.93	19.00	7.12	3.46	0.032
**FFM (%)**	77.31	78.10	6.49	80.52	80.90	6.56	81.07	81.00	7.12	3.46	0.032
**Muscle (%)**	70.30	70.71	6.64	75.06	76.41	7.38	75.60	76.02	7.66	5.82	0.003
**TBW (%)**	55.58	56.00	4.37	58.74	59.10	4.93	58.97	58.90	5.13	5.42	0.004
**Mother**											
**BFP (%)**	23.58	21.40	7.25	19.79	19.10	6.44	18.98	19.20	7.11	2.74	0.065
**FFM (%)**	76.42	78.60	7.25	80.21	80.90	6.44	81.02	80.80	7.11	2.74	0.065
**Muscle (%)**	72.54	74.43	7.21	74.48	76.06	7.26	75.40	75.92	7.82	1.15	0.316
**TBW (%)**	56.52	57.60	4.92	58.29	58.90	4.84	58.94	58.85	5.20	1.67	0.190

BFP—body fat percentage; F—result of the one-way ANOVA test (Fisher’s test); FFM—fat free mass; Me—median; *p*—probability value; SD—standard deviation; TBW—total body water; $\bar{x}$—average value.

**Table 7:** The post-hoc test in reference to the difference between the body build parameters in relation to education of fathers.

Post-Hoc	BFP (%)			FFM (%)			Muscle (%)			TBW (%)		
	Professional	Medium	High	Professional	Medium	High	Professional	Medium	High	Professional	Medium	High
**Father’s Education level**												
Professional		0.064	0.023		0.064	0.023		0.007	0.002		0.013	0.007
Medium	0.064		0.776	0.064		0.776	0.007		0.815	0.013		1.000
High	0.023	0.776		0.023	0.776		0.002	0.815		0.007	1.000	

The values of the probability coefficient for the post-hoc test (Tukey’s test); BFP—body fat percentage; FFM—fat free mass; TBW—total body water.

**Table 8:** Relationship between parents’ BMI and the parameters of the child’s body structure.

Variables	R	*p*
**Fathers**		
Father’s BMI vs. child’s BFP (%)	0.06	0.278
Father’s BMI vs. child’s FFM (%)	−0.06	0.278
Father’s BMI vs. child’s muscle (%)	−0.08	0.138
Father’s BMI vs. child’s TBW(%)	−0.08	0.143
**Mothers**		
Mother’s BMI vs. child’s BFP (%)	0.17	0.003
Mother’s BMI vs. child’s FFM (%)	−0.17	0.003
Mother’s BMI vs. child’s muscle (%)	−0.19	0.001
Mother’s BMI vs. child’s TBW(%)	−0.18	0.001

R—value of Spearman’s rank correlation; *p*—level of probability.

**Table 9:** The relationship between the parents’ body weight category and the parameters of the child’s body composition.

The Parameters of the Child’s Body Composition	Body Mass Category Based on BMI									F	*p*
	Norm			Overweight			Obesity				
	$\bar{x}$	Me	SD	$\bar{x}$	Me	SD	$\bar{x}$	Me	SD		
**Father**											
**BFP (%)**	18.83	18.90	6.74	19.75	19.25	7.00	19.74	19.20	6.75	0.55	0.574
**FFM (%)**	81.17	81.10	6.74	80.25	80.75	7.00	80.26	80.80	6.75	0.55	0.574
**Muscle (%)**	75.92	76.56	7.29	74.49	75.56	7.77	74.62	75.99	7.33	1.08	0.341
**TBW (%)**	59.14	59.25	5.09	58.50	58.75	5.05	57.91	58.70	4.99	1.01	0.364
**Mother**											
**BFP (%)**	18.61	18.65	6.77	21.12	20.45	7.01	19.48	18.85	4.52	4.53	0.011
**FFM (%)**	81.39	81.35	6.77	78.88	79.55	7.01	80.52	81.15	4.52	4.54	0.011
**Muscle (%)**	75.92	76.67	7.43	72.77	72.73	7.77	76.09	76.77	4.65	6.14	0.002
**TBW (%)**	59.20	59.30	5.04	57.40	57.15	5.01	58.82	59.25	3.41	4.35	0.013

BFP—body fat percentage; F—result of the one-way ANOVA test (Fisher’s test); FFM—fat free mass; Me—median; *p*—probability value; SD—standard deviation; TBW—total body water; $\bar{x}$—average value.

**Table 10:** The post-hoc test in reference to the difference between the body build parameters in relation to mother’s body mass category.

Post-Hoc	BFP (%)			FFM (%)			Muscle (%)			TBW (%)		
	Norm	Overweight	Obesity	Norm	Overweight	Obesity	Norm	Overweight	Obesity	Norm	Overweight	Obesity
**Mother’s body mass category**												
Norm		0.007	0.861		0.007	0.861		0.002	0.995		0.009	0.949
Overweight	0.007		0.609	0.007		0.609	0.002		0.189	0.009		0.501
Obesity	0.861	0.609		0.861	0.609		0.995	0.189		0.949	0.501	

The values of the probability coefficient for the post-hoc test (Tukey’s test); BFP—body fat percentage; FFM—fat free mass; TBW—total body water.

## Data Availability

Data available from the authors of this publication.
